# Editorial: The cost of war: sociological approaches to the societal and individual wounds of combat

**DOI:** 10.3389/fsoc.2025.1767848

**Published:** 2026-01-16

**Authors:** Jan Grimell, Hazel Atuel, Cave Sinai

**Affiliations:** 1 Department of Sociology, Faculty of Social Sciences, Umeå University, Umeå, Sweden; 2 University of Southern California, Los Angeles, CA, United States; 3 Center for Disaster Medicine, University of Gothenburg, Gothenburg, Sweden

**Keywords:** combat, conflict, diagnosis, health, illness, reintegration, war, wounds

Today's global situation serves as a reminder that conflict and war, ranging from tribal fights to international hostilities, are seemingly inescapable social facts woven throughout the course of human history (Malešević, 2010). In all their shapes and forms, conflict and war have an existential impact on society, as both individual and collective existence are at stake (Grimell, 2025). The cost of survival can be extremely high and often leaves long-lasting effects, pointing to the depth of actual and potential wounds—from the individual to the societal levels.

The contributions to this Research Topic reflect a wide geographical range, underscoring that the societal and individual costs of war unfold across cultures and nations. The articles are authored by scholars based in, and engage empirically or analytically with, a diverse set of regional contexts, including the Netherlands, Sweden, Finland, Italy, Germany, Austria, the United Kingdom, Northern Ireland/Ireland, Bosnia and Herzegovina, Ukraine, Israel, Australia, and the United States. Taken together, this breadth reinforces the opening assertion that conflict and war are woven throughout human history, while highlighting their enduring and transnational impact across contemporary societies.

Conflict and war inflict both visible and invisible wounds that can negatively affect an individual's health and wellbeing. From our combined research and professional experiences, clinically diagnosable conditions and non-clinical/non-diagnosable illnesses or conditions typically co-exist, with direct consequences to the individual and unintended consequences to their family and their broader community. Post-traumatic stress disorder (PTSD) may be regarded as a prototypical psychiatric and clinical diagnosis. Its precursors became visible in various forms during the First and Second World Wars, and the diagnosis was later formally codified in the Diagnostic and Statistical Manual of Mental Disorders (DSM) in the 1980s (Moss and Prince, 2014). More recent hypothesized constructs have since emerged. Constructs such as moral injury encompass experiences grounded in a distinct etiology (Barr et al., 2022) and possibly also in specific neurobiological processes (Kearney et al., 2023; Lloyd et al., 2021).

Hence, the wounds of conflict and war cannot be understood solely through a clinical lens, as was early recognized by clinicians Shay and Munroe (1998), who worked with U.S. Vietnam veterans; they must also be interpreted through moral and identity-related perspectives (Atuel et al., 2021; Grimell and Atuel, 2023). This call for an interdisciplinary approach has been a longstanding invitation. For as Waller (1944) observed in his study of U.S. veterans from the Revolutionary War, Civil War, and World I, interdisciplinary perspectives are required to address the myriad of challenges surrounding the homecoming journey of veterans. We hold the same view in this Research Topic: divergent viewpoints or conceptual frameworks are needed to convergently grasp the heavy toll of conflict and war, including its profound effects in the deepest layers of human identity and existence. In doing so, we begin to gain a full appreciation of the path back from conflict and war—and the potential healing of visible and invisible injuries.

The mismatch between the complexities and diversities of human experience, on the one hand, and the pursuit of precise scientific solutions, on the other—some of which are profitable—provides fertile ground for an imaginative, progressive, interdisciplinary medical sociological approach (Bradby, 2016).

This Research Topic brings together an international cadre of researchers from sociology, psychology, psychiatry, and other fields engaged in cutting-edge research focused on the costs of war, broadening our understanding of the societal and individual wounds of war.

## A number of articles examine societal support, health care services, sport, surfing- and animal-assisted interventions, and mental health aspects for veterans from a range of perspectives

In *Veteran Welfare Past and Present*, Hopper et al. present a sociological analysis of the Civil War Petitions, illustrating how the social contract has taken shape in both past and present in the UK. Given the structure of today's support systems, new theories must take a certain plurality of support into account.

In their article, Mignogna et al. describe the Veterans Health Administration (VA) Community LeArning CollaboraTive (CO-ACT), which uses a quality-improvement framework and a facilitative process to support community organizations in implementing evidence-based and best-practice suicide-prevention strategies. Community LeArning CollaboraTive includes several components: a toolkit, an organizational self-assessment, a summary of recommendations, the development of a blueprint for change, the selection of suicide-prevention program elements, and an action plan to guide organizations in putting these practices into action.

In *Improving Primary Care Access for Rural Women Veterans*, Cohen et al. developed “Boost Team,” a clinician-driven telehealth outreach service designed to connect these populations with Veterans Health Administration (VHA) services. Their findings show that clinician-led outreach can effectively engage rural cisgender women and gender-diverse Veterans in Veterans Health Administration care, and that there is a clear need for more gender-sensitive services.

In *Understanding the Role of the Invictus Games in Supporting the Transition for UK Disabled Military Veterans*, Mackintosh et al. propose, based on their findings, that promoting sport and physical activity and providing opportunities to participate in sporting events can be an effective tool for supporting veterans. They also offer recommendations for future work on engaging disabled veterans in sport and physical activity, with particular emphasis on the global Invictus Games (IG) movement.

In their article, Castle et al. explore Team Ukraine's experience of taking part in an international sporting event and a 5-week camp for veterans with disabilities and injured active-duty personnel in the UK. Their findings illuminate the challenges the team confronted and overcame, while also providing insight into how competitive sporting events can serve, in times of conflict, both as expressions of national strength and pride and as valuable means of supporting participants' health and wellbeing.

In *Blast Exposure and Long-Term Diagnoses Among Veterans*, Martindale et al. elucidate the longitudinal effects of blast overpressure (BOP) on the brain. Their research underscores the potential for enduring consequences of blast exposure that may necessitate continued engagement with healthcare services well beyond military separation. The findings further suggest that considerations of blast overpressure should inform policy development as well as educational efforts.

In *Wave of Change*, Ossie evaluates the psychological and physiological benefits of surf therapy for US military veterans through the use of wearable technology. The findings demonstrate notable improvements in psychological functioning and underscore the utility of this therapeutic modality, thereby indicating the need for further inquiry into the broader applicability and accessibility of surf therapy for veterans with post-traumatic stress disorder.

In their article on animal-assisted interventions (AAIs) for military families, Nieforth and Leighton suggest that animal-assisted interventions may offer benefits in areas such as communication, relational bonding, and psychosocial wellbeing. Their findings indicate that animal-assisted interventions could serve as an effective complementary intervention for military families.

In their article *Meaning(s) of Transition(s) from Military to Civilian Life at the Intersection with Mental Health*, Misca et al. argue that, in a UK context, it is essential for community services to be aware of the specific needs of veterans. They emphasize that an integrated healthcare system is crucial for supporting military personnel as they transition into civilian life.

In their article on Italian veterans who served in the Balkans, De Angelis et al. employ archival research to investigate long-term mortality and hospitalization among this cohort. The findings—derived from a study characterized by an unprecedented level of follow-up and completeness—do not provide evidence of specific long-term risks of mortality or hospitalization associated with peacekeeping deployments to the Balkans.

In their article on military sexual assault (MSA) and post-traumatic stress disorder among U.S. service members and veterans, Xu et al. suggest that the core symptom network structures of post-traumatic stress disorder may be similar for men and women following military sexual assault. Although men and women show notable differences in their experiences of post-traumatic stress disorder, the structure of the symptom network does not appear to be one of them.

In their article, Livingstone and Blaise found, among other things, that military sexual trauma and lower relationship satisfaction are associated with increased suicide risk among male service members and veterans.

## Several articles highlight how perception, political views, and culture may intersect with veterans' lives

In *Nasty Wars and Needy Veterans*, Phillips examines how perceptions of U.S. deployments shape views of Iraq and Afghanistan veterans as both victims and heroes. The study shows that when deployments were scrutinized, veterans were often cast as naïve victims of a deceptive government, burdened by long-term health issues. This dual hero–victim narrative reflects divergent viewpoints and unequal access to ambiguous information in an increasingly complex society.

In *The 3T Model of Military Veteran Radicalization and Extremism*, situated within a U.S. context, Atuel and Castro examine and delineate the risk factors and protective strategies relevant to mitigating radicalization. As risk factors, three Ts denote the Transmission of Prejudice, Trauma and Adversity, and Transition. As protective strategies, three Ts offer motivations and behavior related to Resistance against Transmission of Prejudice, Addressing Trauma and Overcoming Adversity, and Navigating Transitions.

In their article *The Adaptation of Soldiers to Post-Service Life*, set in an Israeli context, Ben-Shalom et al. argue that political perceptions shape veterans' adaptation by influencing how they make sense of their combat experiences, how they process these experiences individually, and their willingness to continue in reserve service—which in turn provides social support and recognition. Moreover, political perceptions are associated with a sense of bitterness stemming from the decline in public participation in military and reserve service.

In “Unburden Us and Them,” Driessen et al. investigate encounters between Bosnian genocide survivors and Dutch UN veterans. Their findings indicate that, although such encounters hold considerable potential, they are complex undertakings; their success necessitates more sustained attention to, and a deeper understanding of, what meaningful engagement with 'the other' genuinely requires.

In *A Troubling Cost*, Larsson presents a study of republican sacrifice as depicted in murals in Northern Ireland. The article underscores the critical role of visual culture in shaping collective memory and identity, as well as in perpetuating societal hierarchies and cultural violence.

## Four articles highlight the concept of moral injury from a range of perspectives

In two articles on veterans, moral injury, and chaplains in a Dutch context, Mudde et al. demonstrate, first (Mudde et al.(a)), that veterans' potential healing from moral injury involves engaging in moral learning through one-on-one encounters with Dutch military chaplains, and second (Mudde et al.(b)), that military chaplains, through their active presence, create a moral space that enables veterans to explore moral concerns, re-establish self-trust, and recognize the broader sociopolitical dimensions of moral injury.

In *The Prevalence and Long-Term Effects of PTSD and Moral Injury in Swedish Military Veterans*, Nilsson et al. report that the prevalence of moral injury in the studied cohort is low−1.5% based on the cutoff applied.

In his article, Grimell(a) demonstrates that moral injury can be a useful framework for understanding Swedish veterans who are assessed but not diagnosed with post-traumatic stress disorder at the Swedish National Veterans' Clinic, where roughly half of the patients fall into this category—individuals who do not receive a clinical post-traumatic stress disorder diagnosis yet nevertheless experience a marked deterioration in mental health.

## Three articles adopt a distinct identity-focused perspective on veterans' lives

In *Fallout*, Grant et al. investigate the psychosocial harms associated with negative military discharge experiences within an Australian context. Their findings indicate that veterans incur substantial harm when they interpret their discharge as an institutional or personal transgression. Such events—irrespective of their severity—undermine veterans' shared military identity and values, constitute a psychological threat to their sense of belonging, disrupt familial-like bonds, and engender feelings of rejection, diminished self-worth, isolation, and betrayal.

In the article *The Mask of the Warrior*, Grimell(b) suggests that certain components of military identity may prevent or hinder veterans from seeking medical support. These traits may be beneficial during active duty to get the job done, but they are far less helpful in post-service life. Consequently, when deteriorating mental health goes untreated, it may reflect deep-seated health vulnerabilities embedded within veteran identities.

In *You Can Take a Person Out of the Military, but You Can't Take the Military Out of the Person*, Grimell(c) elaborates on the findings of a 10-year identity study on the transition from military to civilian life. Most notably, morality (what is considered right and wrong) appears to have regained strength 10 years after the transition, suggesting that loyalty to military moral frameworks remains more important than adherence to civilian moral regimes.

## Two articles highlight how earlier experiences during deployment can, in various ways, affect life many years later

In *Predisposed Vulnerabilities and Survival among Finnish Soldiers of the Second World War*, Kivimäki et al. present a methodological paper with historical life-course approach—combining quantitative and qualitative analyses—taking into account cultural and sociological contexts within the war is fought, to examine soldiers' predispositions to experiencing war-related violence and stress, and to understand how they responded to it.

In their article *Impact of Life-Threatening Military Incidents During Deployments Abroad on the Relationships Between Military Personnel and Their Families*, Wesemann et al. suggest that, in the German context, such incidents have a substantial impact on the quality and stability of service members' partner and family relationships.

In sum, this Research Topic, *The Cost of War*, is enriched by the inclusion of articles spanning several interconnected themes, examined from a variety of disciplinary perspectives. A number of contributions examine societal support, health care services, sport, surfing- and animal-assisted interventions, as well as mental health dimensions of veterans' lives, illustrating the breadth of factors that shape wellbeing after military service. Several articles shed light on how perception, political views, and cultural contexts intersect with veterans' post-military experiences, underscoring the importance of the social environment in shaping meaning-making and adaptation. Furthermore, multiple studies explore the concept of moral injury from different analytical angles, offering deeper insight into the ethical, emotional, and existential wounds that may accompany military service.

In addition, three articles adopt a distinct identity-focused perspective, revealing how military identity continues to influence veterans long after their transition to civilian life. Two further contributions demonstrate how experiences during deployment can reverberate far into the future, shaping veterans' lives many years later in ways that are both visible and subtle.

Importantly, the contributions to this Research Topic also reflect a broad geographical scope. The articles are authored by scholars based in, and empirically or analytically connected to, a diverse range of regions, including the Netherlands, Sweden, Finland, Italy, Germany, Austria, the United Kingdom, Northern Ireland/Ireland, Bosnia and Herzegovina, Ukraine, Israel, Australia, and the United States. This geographic breadth reinforces the premise introduced at the outset of this editorial—that conflict and war are woven throughout human history—while also underscoring that their consequences are neither culturally nor nationally bounded.

Taken together, these perspectives substantially deepen our understanding of the cost of war. This is, of course, a topic far too vast to be exhausted within a single Research Topic, but we hope that the articles gathered here help address important gaps, enrich understanding in key areas, and illuminate the profound complexity of life after war and conflict across societies.
